# PHREND^®^—A Real-World Data-Driven Tool Supporting Clinical Decisions to Optimize Treatment in Relapsing-Remitting Multiple Sclerosis

**DOI:** 10.3389/fdgth.2022.856829

**Published:** 2022-03-11

**Authors:** Stefan Braune, Elisabeth Stuehler, Yanic Heer, Philip van Hoevell, Arnfin Bergmann, NeuroTransData Study Group

**Affiliations:** ^1^NeuroTransData, Neuburg an der Donau, Germany; ^2^PwC Data and Analytics, Zurich, Switzerland

**Keywords:** multiple sclerosis (MS), personalized medicine, disease modifying agent, real word data, treatment, effectiveness

## Abstract

**Background:**

With increasing availability of disease-modifying therapies (DMTs), treatment decisions in relapsing-remitting multiple sclerosis (RRMS) have become complex. Data-driven algorithms based on real-world outcomes may help clinicians optimize control of disease activity in routine praxis.

**Objectives:**

We previously introduced the PHREND^®^ (Predictive-Healthcare-with-Real-World-Evidence-for-Neurological-Disorders) algorithm based on data from 2018 and now follow up on its robustness and utility to predict freedom of relapse and 3-months confirmed disability progression (3mCDP) during 1.5 years of clinical practice.

**Methods:**

The impact of quarterly data updates on model robustness was investigated based on the model's C-index and credible intervals for coefficients. Model predictions were compared with results from randomized clinical trials (RCTs). Clinical relevance was evaluated by comparing outcomes of patients for whom model recommendations were followed with those choosing other treatments.

**Results:**

Model robustness improved with the addition of 1.5 years of data. Comparison with RCTs revealed differences <10% of the model-based predictions in almost all trials. Treatment with the highest-ranked (by PHREND^®^) or the first-or-second-highest ranked DMT led to significantly fewer relapses (*p* < 0.001 and *p* < 0.001, respectively) and 3mCDP events (*p* = 0.007 and *p* = 0.035, respectively) compared to non-recommended DMTs.

**Conclusion:**

These results further support usefulness of PHREND® in a shared treatment-decision process between physicians and patients.

## Introduction

Shared clinical decision processes in multiple sclerosis (MS) require multidimensional, complex interactions between physicians and patients. There is an asymmetry in knowledge between professionals and laymen regarding available MS therapies, and it is difficult for physicians to clearly convey differences between different treatment options to patients during the limited time of the medical practice visit. This can create diverging treatment expectations between patients and physicians (1–3), impair shared decision processes, and hinder necessary adherence. Personalized data driven clinical prediction tools with informative visualization can facilitate these discussions and improve the joint doctor-patient efforts to implement the individually most effective DMT, yet no such tools were available in the past for routine use.

One barrier to the development of efficient decision-support tools is a lack of relevant data sources. Although the number of choices of different disease-modifying therapies (DMTs) for relapsing-remitting MS (RRMS) with diverse treatment mechanism increases, information from randomized clinical trials (RCTs) in RRMS usually remains limited to a single head-to-head study with one of the available DMTs. The total observation time within such trials is usually 2 years, and no information can be derived regarding next-best treatment options or allowing for patients' preferences. The RCT's two active arms perspective thus provides only limited information for overall longer-term treatment options and more complex treatment requirements in an individual patient.

Real-world data (RWD) and advanced statistical methods are utilized in growing numbers of comparative effectiveness studies aiming to fill this gap (4–8), but also these efforts remain confined to a retrospective cohort view and are not suitable to support personalized decision strategies for clinical routine.

An alternative idea is to base models on “objective” measures and clinical predictors, such as the biomarker neurofilament light chain. This marker has indeed enabled first insights into probable dynamics of relapsing remitting multiple sclerosis (RRMS) also from a cohort perspective [for review see (9)]. It has also shown predictive potential when measured as a *post-hoc* response marker to DMTs (10). In addition, enzyme-linked immunospot assay (ELISPOT) testing of B-cell activity has been shown to successfully predict the likelihood of individual DMT responsiveness to interferons or glatiramer acetate (11). Despite these advances, it appears highly unlikely that single or sets of biomarkers will become available in the foreseeable future to support personalized treatment decisions in all MS patients and for all DMTs.

To meet the multiple demands of improved communication between patients and doctors and data-driven decision making based on real-world experience, NeuroTransData (NTD) and PricewaterhouseCoopers (PwC) embarked on the development of a mathematical algorithm based on real-world data from the NTD MS registry to calculate the probabilities of patients with RRMS in diverse clinical situations to remain free of relapse and free of 3 months confirmed disability progression (3mCDP) for available DMTs.

A previous publication provided comprehensive information on methods, validity and robustness of the predictive models implemented in the web-based tool called “Predictive Health Care with Real-World Evidence in Neurological Disorders” (PHREND^®^) (12).

In brief, we implemented two hierarchical Bayesian generalized linear models (GLMs) to predict the probabilities of (a) freedom of relapse activity, (b) freedom of 3 months confirmed disability progression (CDP) for every of the currently available DMTs after a switch from a previous DMT. The predictive framework was based on RWD collected in the NTD MS registry consisting of clinical data including patient characteristics and disease history. Predictors used for the predictive models are: age, gender, duration of RRMS, previous therapy and its duration, indicator if one of the two previous therapies was second line, EDSS total score, number of relapses within last 12 months, time since last relapse. Based on these individual information probabilities for both effectiveness parameters can be calculated for a prospective period up to 4 years, scalable at the discretion of the user.

Assessment of the model performance demonstrated that both models provided robust and accurate predictions and that both models generalized to new patients and clinical sites. The predictive relapse model achieved an average out-of-sample C-Index of 0.65 and an average out-of-sample mean squared error (MSE) of 0.76 relapses. The predictive CDP model achieved an average C-Index of 0.58 and an average out-of-sample MSE of 0.12 CDPs. Robustness against different choices of priors was proven by the fact that changing the prior distributions did not influence the predicted therapy ranking.

Accounting for individual clinical patient characteristics, the resulting predictive probabilities are intended to be provided during the discussion of potential change in DMT to support the shared decision process between physicians and patients.

We herein report on the clinical value and further validation of PHREND® with three new sets of results: (1) update of the models' performance over time, after more data of the ongoing NTD MS registry collection has been added regularly to re-train the models since the initial publication in 2020 (12), which was based on a data cut from 2018; (2) external validation by comparing of PHREND^®^ predictions to RCT results based on current models, including new DMTs which have entered the German treatment landscape since the last data cut and were subsequently integrated into the training sets of the model; (3) new assessment of clinical relevance of the recommendations based on whether patients received DMTs recommended by PHREND^®^ or not.

## Materials and Methods

### Database and Parameter

#### NTD MS Registry

This study employed real-world data recorded in the NTD MS registry. NTD is a Germany-wide network of physicians in the fields of neurology and psychiatry that was founded in 2008. Each practice is certified according to network-specific and ISO 9001 criteria. Compliance with these criteria is audited annually by an external certified audit organization.

Codes uniquely identifying patients are managed by the Institute for Medical Information Processing, Biometry and Epidemiology [Institut für medizinische Informationsverarbeitung, Biometrie und Epidemiologie (IBE)] at the Ludwig Maximilian University in Munich, Germany, acting as an external trust center. Written informed consent is obtained from each patient providing data for the MS registry. The data acquisition protocol described above was approved by the ethical committee of the Bavarian State Medical Association (Bayerische Landesärztekammer; June 14, 2012, ID 12114) and re-approved by the ethical committee of the Medical Association of North-Rhine (Ärztekammer Nordrhein; April 25, 2017, ID 2017071).

Demographic and clinical parameters of MS patients are captured in real time with an average of 3.7 Expanded Disability Status Scale (EDSS) assessments per patient year. Data quality is monitored by the NTD data management team, and data inputs are checked for inconsistencies and errors manually and by using an automated error-analysis program. Additionally, automated and manually executed queries are implemented to check for inconsistencies and request missing information. All data are pseudonymized and pooled to form the NTD MS database.

### Data Extraction Period, Numbers of Patients

The current study is based on the same original dataset as described in the previous publication (12) and additional quarterly data cuts extracted from the NTD MS registry between July 1, 2018 and October 2020. After quality control and application of inclusion criteria as described (12), this dataset includes 3,119 patients.

### Predictive Models and Selection of Predictors

PHREND® supports treatment decisions for optimization of treatment switches from a current DMT in RRMS, which needs to be discontinued due to lack of efficacy or adverse events, to another therapy. The minimum time between diagnosis of MS and first application of PHREND^®^ must be 6 months. Because the EDSS scale is not linear across its entire range from 0 (“normal”) to 10 (“death due to MS”), PHREND^®^ cannot be used if the current EDSS is higher than 6. Based on individual patient characteristics at the time of the intended switch, PHREND® provides predictive probabilities to remain free of relapse and free of EDSS-based 3mCDP under the newly chosen DMT. The probability of staying relapse-free is derived from modeling the number of relapses following a negative binomial distribution and subsequently computing the fraction of predicted count of relapses that equals zero. The probability of staying 3mCDP-free is derived from modeling it as a binary event. Both models account for varying observation time in the training set [i.e., for varying time on DMT (12)]. Information used for the calculations comprises age, gender, EDSS, current therapy and duration of current therapy, number of previous DMTs and information if one of those was already a second-line treatment, time since RRMS diagnosis, number of previous relapses in the last year, and time since the last relapse. The choice of these parameters as predictors was based (1) on availability of sufficient data to train the statistical models on a representative population, (2) routine collection in clinical practice as prerequisite for widespread usability, and (3) proof of impact strength and usefulness of each parameter on the prediction (12).

### Internal Validation and Prediction Quality Over Time

Underlying considerations, methods and results of first internal validations of PHREND® were previously communicated (12). Here, the mean square error (MSE) as well as the negative log-likelihood (NLL) are used to assess the goodness-of-fit of the models (i.e., the deviation between observed and predicted outcomes). The C-Index (0 to 1, where “1” indicates perfect predictions) measures the discrimination accuracy of a model and is defined as the proportion of concordant pairs (i.e., predicted outcomes match actual outcomes) divided by the total number of possible evaluation pairs. All three of these measures are computed in-sample and out-of-sample, where either predictions for patients used for training the models or for a set of new and unseen patients are evaluated to understand the model's generalizability. The credible intervals of the resulting model coefficients are indicators for the prediction certainty (i.e., the smaller the more certain the prediction). They are computed empirically, where a large set of models are fitted based on a randomly sampled initialization, and subsequently the range of each coefficient is described by credible intervals using the 90%-intervals from the resulting sets of coefficients. A small interval shows that, despite randomly selected initial values, the observed patient data for training is sufficiently informative to produce similar coefficients.

Because PHREND® is based on data extracted from the routinely used ongoing NTD MS registry (13), there is a steady increase of information (~1.3% increase of patient numbers per quarter in the year 2020, data not shown here). Therefore, PHREND® is updated on a quarterly basis and prediction quality over time is monitored. For this work, the performance measures as described above were calculated repeatedly for models trained on quarterly updates of the database up to and including October 1, 2020.

### External Comparison With RCTs

For external comparisons, PHREND® predictions were compared to results of RCTs to assess consistency with current research results. For this analysis, the published results of the active treatment cohorts of the clinical trials CONFIRM [dimethyl fumarate, glatirameracetate (14)], DEFINE [dimethyl fumarate (15)], REGARD [interferon-ß, glatiramer acetate (16)], TRANSFORMS [interferon-ß, fingolimod (17)], AFFIRM [natalizumab (18)], CLARITY [cladribine (19)], OPERA I and II [ocrelizumab (20)] and TEMSO [teriflunomide (21)] were chosen. For each study population, a comparable cohort in the NTD MS registry was identified by aiming to apply the same inclusion criteria as described in the corresponding study and by subsequently comparing mean values and standard deviations of continuous and distributions of categorial parameter to assure comparability of clinical and demographic baseline characteristics between groups.

Probabilities of staying free of relapses and free of 3mCDP within the clinical study timeframe were predicted using PHREND® models for the corresponding NTD MS cohorts, and their means and 90% credible intervals were compared to published results for each active treatment arm.

### Clinical Robustness and Value

PHREND® provides personalized ranking of predictions for all DMTs available in Germany, which are ordered with respect to either highest probability for the patient to be relapse- or 3mCDP-free (**Figure 5**). The clinical usefulness and superiority of outcome of these recommendations made by PHREND® was previously affirmed by comparing therapy effectiveness for selected DMT cohorts where the recommended DMT was prescribed vs. where another DMT was chosen (12).

The current analysis investigates three different scenarios based on all DMTs simultaneously and independently of which substance was ranked highest or lowest: (1) patients taking the highest ranked therapy, (2) patients who took one of the two highest ranked therapies, and (3) patients who took one of the two least ranked therapies, always contrasted with results from patients on any other of the lower or higher-ranked treatments, respectively.

The comparability of patient groups in the analyses is ensured by a preceding propensity-score-based weighted matching (22) based on defined patient characteristics (age, time since diagnosis, previous DMT, duration of previous DMT, number of previous DMTs, indication if one of the previous DMTs was a second line treatment, gender, EDSS, time since last relapse, number of relapses in the last year). Relapse activity and 3mCDP is plotted for both subgroups in boxplots.

### Implementation of the PHREND® Algorithm in a Web-Based Application

The web-based PHREND® application was developed using a human-centered-design approach over ten design iterations, in direct collaboration with doctors and patients to provide a clear, intuitively understandable presentation of the calculations for each DMT and the robustness of each probability. The presentation needs to integrate options to reflect upcoming questions in the shared decision process regarding their impact on choices between DMTs. PHREND® can be used as a standalone solution with clinical data being entered manually per patient or as part of the patient management platform DESTINY^®^ (13) with automated data transfers.

## Results

### Internal Validation and Prediction Quality Over Time

**Figure 1:**
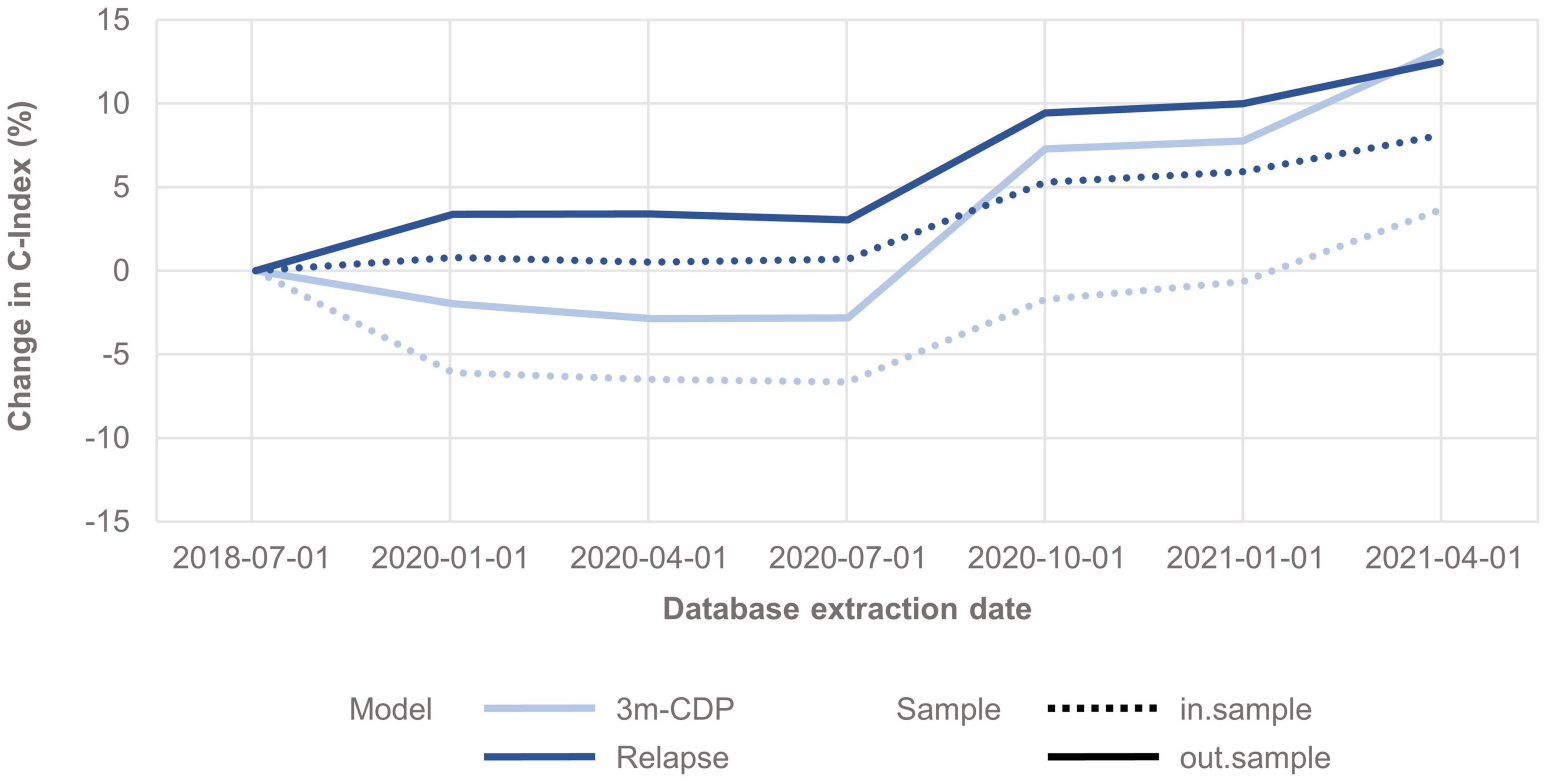
Change in discrimination accuracy over time (C-Index) for the 3mCDP and the Relapse model, with the model performance based on the data extract from July 2018 as reference point. The plots show an increase of discriminative performance over time for the relapse model, whereas the performance of the CDP model initially dropped and only recovered during the last quarters analyzed for this work. Dashed lines show the performance change for the in-sample predictions, i.e., the predictions for patients used for training the model, and solid lines show the performance change when predicting for unseen patients, i.e., they address how well the model generalizes to an unknown population.

The analysis shows that the span of credible intervals of the models' coefficients decreased consistently over time (Supplementary Figures 1, 2). The discrimination accuracy (C-Index) of the models over time [with the published performance (12) as a reference point] increased for the relapse model for both in-sample and out-of-sample predictions (Figure 1). After an initial decrease, the discrimination accuracy for the 3mCDP model also increased with the availability of new data, out-performing the initially published model in the last quarter (out-of-sample). The apparent increase of the performance in Oct 2020 was driven by the inclusion of new treatments ocrelizumab and cladribine, due to short observation time and informative priors used for training the models. This effect was observed to even out in the subsequent quarters.

### Comparison With External RCT Data

**Figure 2:**
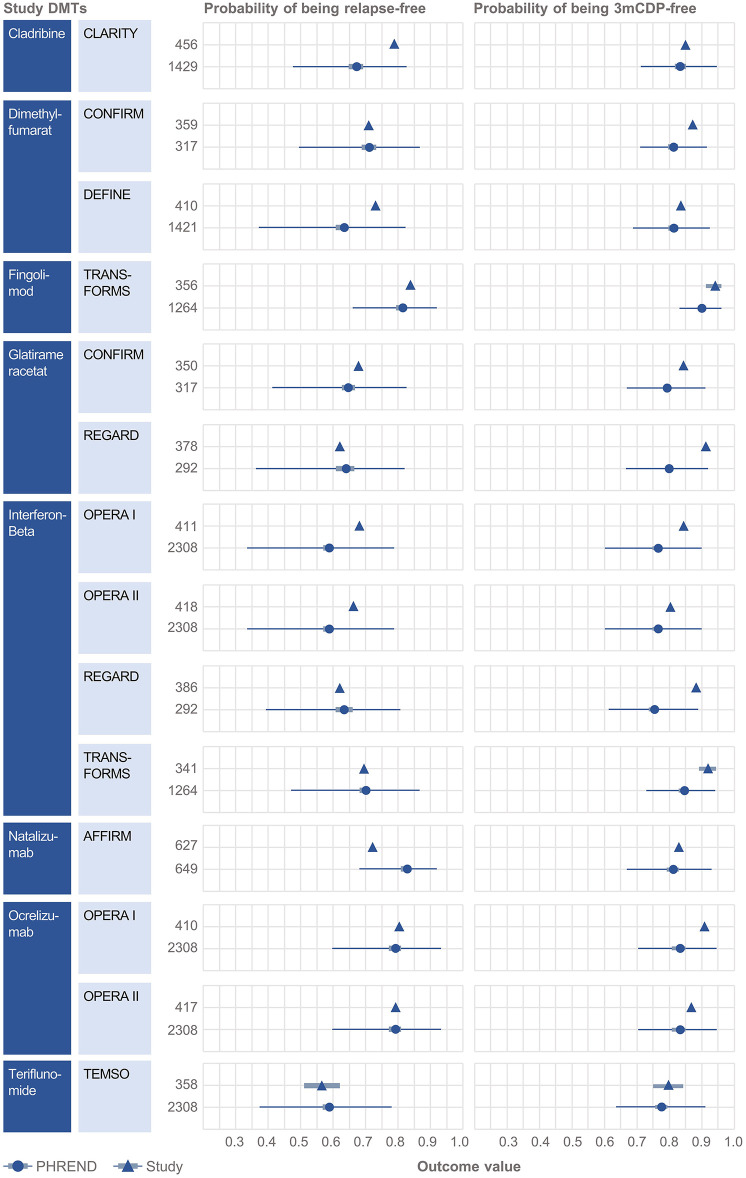
External validation of the PHREND models using outcomes from eight clinical studies. A black triangle denotes the mean outcome reported by the clinical study, and the bar its 90%-confidence interval (if available). In analogy the PHREND model results are shown in red dots, with additional information on range of predictions. The wider bars show the 90%-confidence interval of the prediction. The thin horizontal bars show the range between the 5 and 95% quantile of all predictions. The numbers represent the patients included into the respective analysis. CONFIRM (14), DEFINE (15), REGARD (16), TRANSFORMS (17), AFFIRM (18), CLARITY (19), OPERA I and II (20) and TEMSO (21).

Clinical baseline characteristics (MS duration, age, EDSS, relapses within previous 12 months, time since last relapse) were highly consistent between the NTD MS registry and RCT study populations (Supplementary Figures 3, 4). Probabilities predicted by PHREND® for being relapse free and 3mCDP free mostly approximated the results reported by the corresponding clinical study (Figure 2). Almost all differences were smaller than 10% between the predicted and the real study results, with the exemption for relapse activity with cladribine [CLARITY (19)], and 3mCDP with glatiramer acetate [REGARD (16)] and interferon-ß [REGARD (16)].

### Clinical Consistency and Value

**Figure 3:**
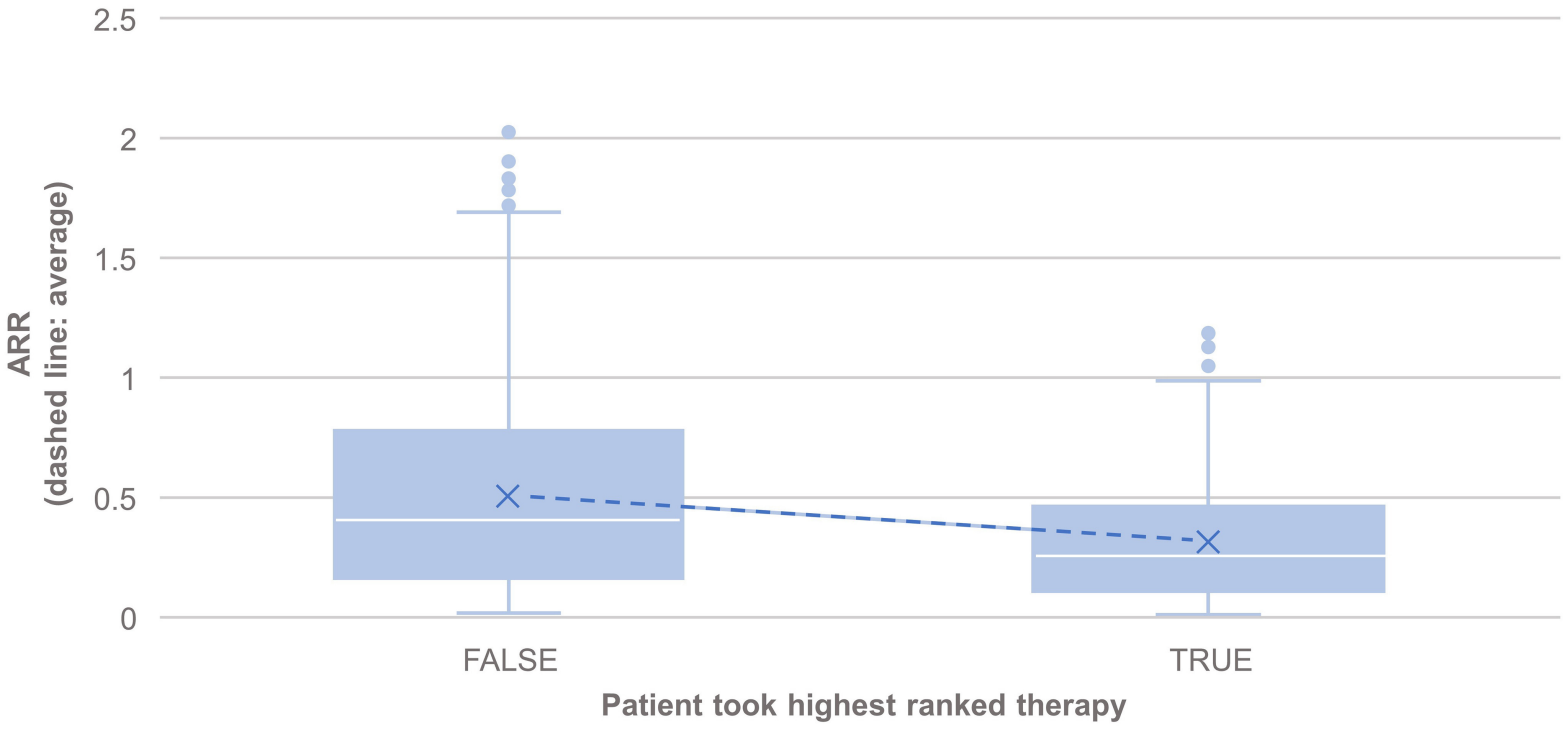
Comparison of the annualized relapse rate after propensity score matching of groups for the relapse model, based on the disease courses as observed in the registry. *N* = 495 of 3,119 patients took the highest ranked DMT recommended by PHREND (case “TRUE”). ARR was significantly lower in this group compared to patients, who did not follow the reconditions and chose another DMT (“FALSE, *N* = 2,624”, *p* < 0.001). The blue points show mean ARR for each subgroup, with a line to visualize the resulting slope.

PHREND® provides a real-world data-driven ranking of therapies for both endpoints (see **Figure 5**). A the group level, the probability of staying relapse-free after propensity-score based weighting was statistically significantly higher (*p* < 0.001) for patients taking the highest-ranked therapy as opposed to any other of the lower ranked treatments (Figure 3).

**Figure 4:**
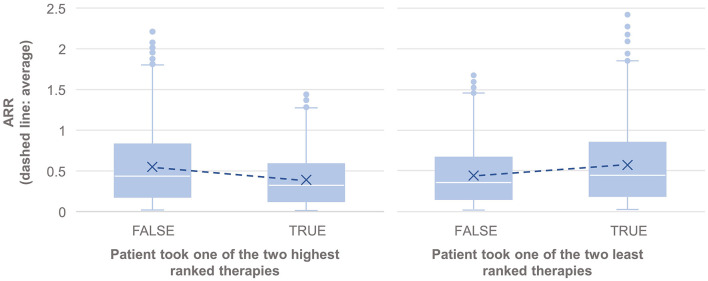
Comparison of the annualized relapse rate after propensity score matching of groups for the relapse model, based on the disease courses as observed in the registry. *N* = 1,165 of 3,119 patients took one of the first two DMTs recommended by PHREND (left), and *N* = 1,076 of 3,119 patients took one of the two least ranked DMTs (right). The blue points show the mean ARR for each subgroup, with a line to visualize the resulting slope in comparison to patients with other DMT decisions. ARR was statistically significantly lower when following the recommendation of the two highest ranked therapies (left, *p* < 0.001), and significantly higher for patients on one of the two least ranked DMTs (right, *p* < 0.001).

The results also showed statistically significantly lower annual relapse rate (ARR) in patients who received one of the two highest ranked DMTs as recommended by PHREND® (*n* = 1,954, *p* < 0.001), and statistically significantly higher ARR in patients who had received one of the two least-recommended DMTs (*n* = 2,043, *p* < 0.001) (Figure 4). Statistically significant superiority was also found when the effects of personalized DMT selection were evaluated for the risk of CDP (Table 1).

**Table 1:** Comparison of therapy effectiveness for relapse and 3mCDP models in propensity score matched patient groups receiving DMTs recommended by PHREND vs. other than recommended DMTs.

DMT^*^	**Model**	**Slope coefficient^**a**^**	Sample size treated with recommended DMT^*^	**Sample size** treated with other than recommend DMT^*^	***p*** **-value**
Highest ranked DMT	Relapse	−0.5193	495	2,624	<0.001
Highest ranked DMT	3mCDP	−0.4544	570	2,549	0.007
First or second highest ranked DMTs	Relapse	−0.4130	1,165	1,954	<0.001
First or second highest ranked DMTs	3mCDP	−0.2377	1,191	1,928	0.035
One of the two least ranked DMTs	Relapse	0.3695	1,076	2,043	<0.001
One of the two least ranked DMTs	3mCDP	0.3018	952	2,167	0.009

a *Derived from a survey-weighted negative binomial generalized linear model (Relapse) or a survey-weighted binomial generalized linear model (3mCDP), where negative sign indicates lower disease activity*.

* *DMT, one or more disease modifying therapies that were ranked according to description*.

### Implementation of the PHREND® Algorithm in a Web-Based Application

**Figure 5:**
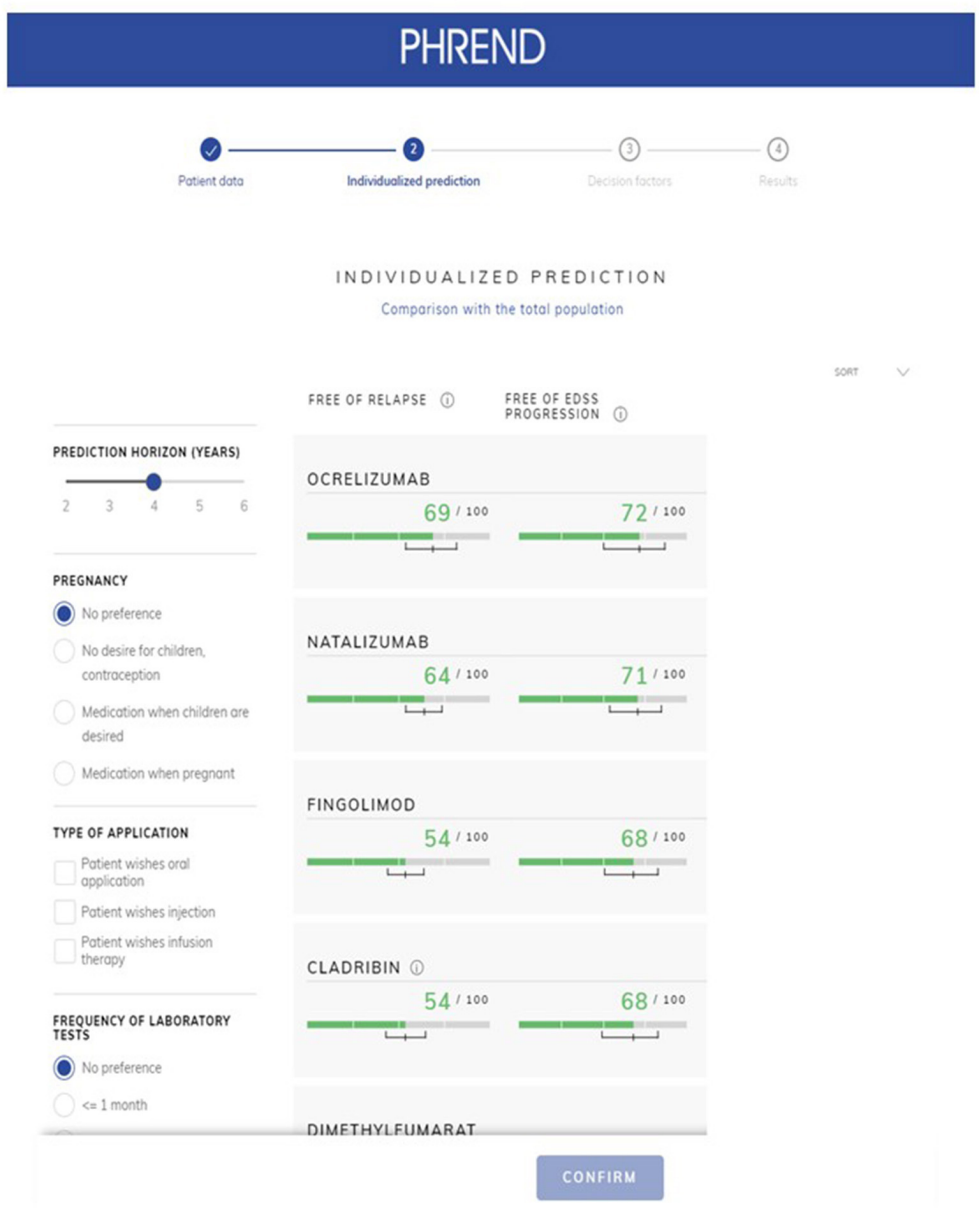
PHREND display of resulting personalized predictions. The green horizontal bars show the individual probability of staying free of relapse (left column) or free of 3mCDP (right column) in the chosen time period (upper left corner, here 4 years). Additionally, a 90% credible interval is displayed for each prediction, i.e., the outcome is within this range with 90% probability. Disease modifying therapie's generic names are displayed in the application.

Probabilities of outcomes under each available DMT are graphically displayed as natural frequencies in a ranked manner according to the results of the predictive calculations (Figure 5). This presentation of the probabilities corresponds to the current state of research in medical communication and was tested to be well understood by physicians and patients. The length of the prediction period can be chosen between 2 and 6 years. 90% credible intervals are displayed for each prediction to provide information on the homogeneity of the single results and to demonstrate possible overlap between outcomes. The smaller the interval, the more reliable is the prediction for the individual patient. To support the workflow of the shared decision process between physicians and patients, personal preferences such as family planning, route of administration and others can be incorporated. In these cases, not-suitable DMT options are shaded to allow the demonstration of the impact of certain preferences on the available DMT spectrum to choose from and the possible consequences regarding effectiveness of treatment options.

**Figure 6:**
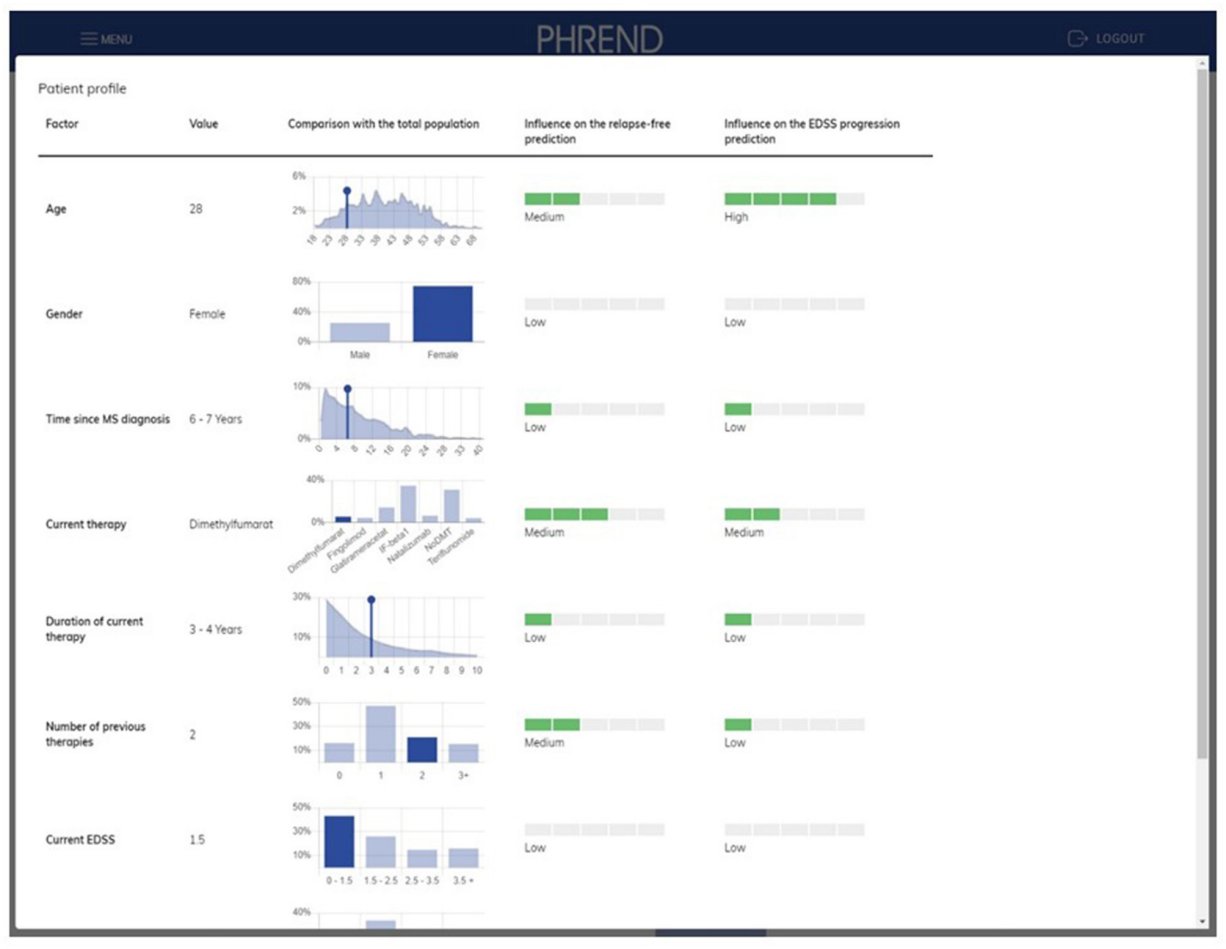
PHREND display of parameter distribution and impact of biographic and medical factors on prediction. On the left side, the patient's personal characteristics are shown within the distribution derived from the whole patient population. On the right side, the impact of each characteristic of the patient for the personalized prediction of being relapse-free and disability progression-free is shown.

On-demand, deeper insights on the factors contributing to a single prediction are provided on an extra page (Figure 6). Results can be stored as PDF file as hand-out for the patient and for documentation purposes in medical record systems.

## Discussion

The previously published, initial assessment of the model performance demonstrated mathematically robust and accurate predictions based on the C-index and MSE (12). Models predicting freedom of relapse and of 3mCDP were shown to generalize to new patients and clinical sites and were robust against different choices of the priors and against sample size. In the current work, the same measures were analyzed based on quarterly updated database extractions, demonstrating the robustness of the models' predicted effectiveness probabilities with about 1.3% per quarter new patient data over time and also after the addition of the new DMTs cladribine and ocrelizumab. In parallel with this evidence of increasing accuracy of the models, the reliable performance of the model is demonstrated herein though consistently decreasing spans of credible intervals of the models' coefficients over time. These observations underscore the essential necessity for ongoing monitoring of the database and provide example metrics to ensure the models' performance and consistency. With continued application of the routines described herein, the beneficial effect of increasing data on the quality of the predictive probabilities can be expected to continue.

In an additional step toward external validation, this study showed that differences were smaller than 10% between predicted probabilities of PHREND® for freedom of relapse activity and 3mCDP, respectively, in NTD MS registry real-world patient cohorts compared with results from prospective RCT cohorts in a total of 14 active arms derived from 8 RCTs. The evident similarity of predictions of PHREND® based on real-world data and the published results from prospectively captured, blinded data from RCTs provide a meaningful external confirmation of the robustness of the algorithms employed. Observed differences of the comparisons in relapse activity in the CLARITY study (19) and 3mCDP in OPERA (20) and REGARD (16) trials likely reflect differences in patient characteristics not captured in the available cohort information, because pairwise single-patient based matching was not possible. For example, CLARITY (19) included 75% treatment-naïve patients, while all patients from the NTD MS registry were switching DMTs. Additional external validations are planned to further explore the validity of PHREND® in an ongoing endeavor to understand and improve predictive accuracy.

When comparing patients who, based on a combination of clinical consideration and individual preferences, chose the highest-ranked, or one of the two top-ranked DMTs, with patients who did not, the clinical course in both approaches was statistically significantly better regarding frequency of relapse activity and 3mCDP than in the comparator group with lower-ranked DMTs. Conversely, the opposite effect was shown with statistically significantly negative effects on both effectiveness parameter, if one of the two lowest-ranked DMTs was chosen compared to the top-ranked DMTs by the algorithm. This demonstrates the real-world accuracy of the mathematical algorithm developed in identifying the optimal DMTs in individual patients based on patient-specific parameters and real-world practice situations. Further validation is necessary with external non-NTD personalized patient data to evaluate robustness and generalizability of the algorithm to other datasets.

It is important to note that PHREND® does not intend to automatize the medical decision process but to provide additional information beyond cohort-based study results and intuition. Integrated into the complex shared-decision process, it empowers physicians and patients to select optimal DMTs individually by providing data-driven, quantified outcome probabilities. Initial feedback obtained from NTD clinicians and patients indicate that the integration of PHREND® into the shared-decision process results in a more structured, rational communication process, which can reduce fears and avoidance patterns in patients and provide a base for a time-efficient shared-decision process. It remains to be evaluated, how this experience of a personalized transparent therapy decision can contribute to patients' motivation and adherence. Mandatory CE certification for PHREND® as medical tool is currently being obtained. Registration for PREND® is restricted to physicians, because it is an integral part of a medical process (https://www.neurotransdata.com/en/destiny#phrend).

## Data Availability Statement

The raw data supporting the conclusions of this article will be made available by the authors, without undue reservation.

## Ethics Statement

The studies involving human participants were reviewed and approved by Bavarian Medical Board (Bayerische Landesärztekammer; June 14, 2012, ID 12114), Medical Board North-Rhine (Ärztekammer Nordrhein; April 25, 2017, ID 2017071). The patients/participants provided their written informed consent to participate in this study.

## Author Contributions

SB: concept, analysis, and interpretation and writing. ES: concept, data preparation, analysis, and interpretation and writing. YH: analysis and interpretation. PH: concept, interpretation, supervision, and project administration. AB: concept, project administration, and funding. All authors contributed to the article and approved the submitted version.

## Funding

This work was funded by NeuroTransData GmbH, Neuburg/Donau, Germany and PricewaterhouseCoopers, Zurich, Switzerland. The commercial entities of NeuroTransData and PricewaterhouseCoopers had no role in study design, data collection and analysis, decision to publish, or preparation of the manuscript.

## Conflict of Interest

SB, AB, and all members of the NeuroTransData Study Group are working self-employed as medical doctors in outpatient practices. All of them are members of the NeuroTransData doctors' network (https://www.neurotransdata.com/en/about-us). SB has received honoraria from Kassen4rztliche Vereinigung Bayern and HMOs for patient care; honoraria for consulting, project management, clinical studies, and lectures from Biogen, CSL Behring, NeuroTransData, Novartis, Roche and Thieme Verlag; honoraria and expense compensation as board member of NeuroTransData. AB has received consulting fees from advisory board, speaker, and other activities for NeuroTransData; honoraria and expense compensation for project management and clinical studies from Novartis and Servier. ES, YH, and PH are employees of PricewaterhouseCoopers, Z, Switzerland.

## Publisher's Note

All claims expressed in this article are solely those of the authors and do not necessarily represent those of their affiliated organizations, or those of the publisher, the editors and the reviewers. Any product that may be evaluated in this article, or claim that may be made by its manufacturer, is not guaranteed or endorsed by the publisher.
