# Supplementary material for: PHREND®—A Real-World Data-Driven Tool Supporting Clinical Decisions to Optimize Treatment in Relapsing-Remitting Multiple Sclerosis
Source: Front Digit Health. 2022 Mar 11;4:856829. doi: 10.3389/fdgth.2022.856829 (PMC8961981; doi:10.3389/fdgth.2022.856829)
Supplement: Supplementary file 1 [file Table_1.docx]

**Supplement information**

1. **PHREND prediction quality over time – credible intervals**

As PHREND is based on the data extracted from a registry that is routinely used by doctors in day-to-day practice, there is a steady increase of information on patients and their treatment (approx. 1.3% increase of patients per quarter was observed in the year 2020). Therefore, PHREND is updated on a quarterly basis and prediction quality over time is monitored.

With the availability of new data, it is expected that the precision of PHREND’s predictions increases, and indeed it can be seen that the span of credible intervals of the models’ coefficients decreases consistently over time. The smaller this span gets, the more precise the predictions of PHRENDs become, i.e. the overall credible intervals become smaller and the predictions therefore more meaningful. Supplementary figures 1 and 2 show this development of the coefficients in detail for the relapse and the CDP model, respectively.

**Supplementary Figure 1:** Span of model coefficients’ credible intervals over time for the relapse model. Almost all credible intervals are consistently decreasing, implying that the predictions of PHREND are becoming more precise with the inclusion of more patient data & therapies.

**Supplementary Figure 2:** Span of model coefficients’ credible intervals over time for the 3mCDP model. Almost all credible intervals are consistently decreasing, implying that the predictions of PHREND are becoming more precise with the inclusion of more patient data & therapies.

1. **Population comparisons for external validation between RCT patient cohorts and matched NTD MS cohorts being treated with the same DMTs.**


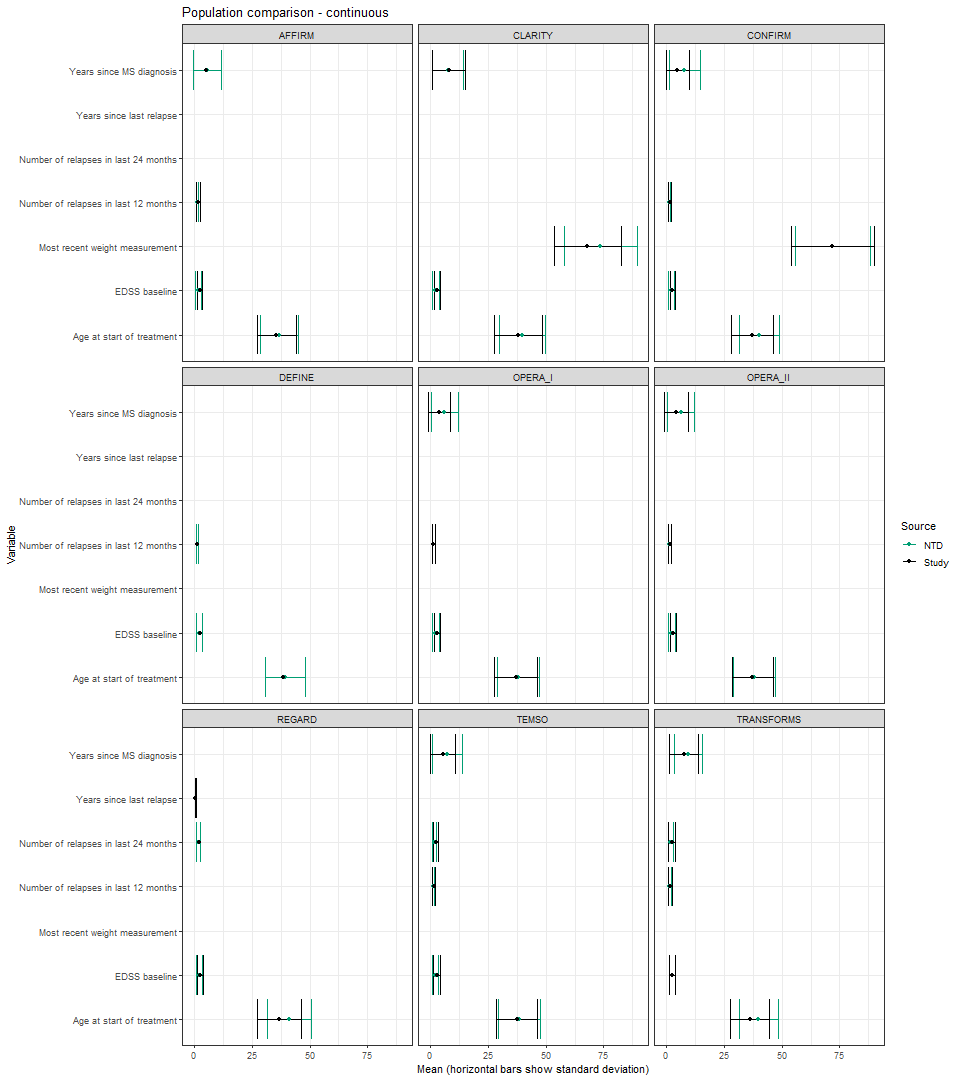


**Supplementary Figure 3:** Comparison of external study cohort and matched NTD MS registry cohort: continuous patient characteristics. They match very closely, with only some difference in mean age, i.e. the NTD populations tend to be a bit older on average than the corresponding study populations.


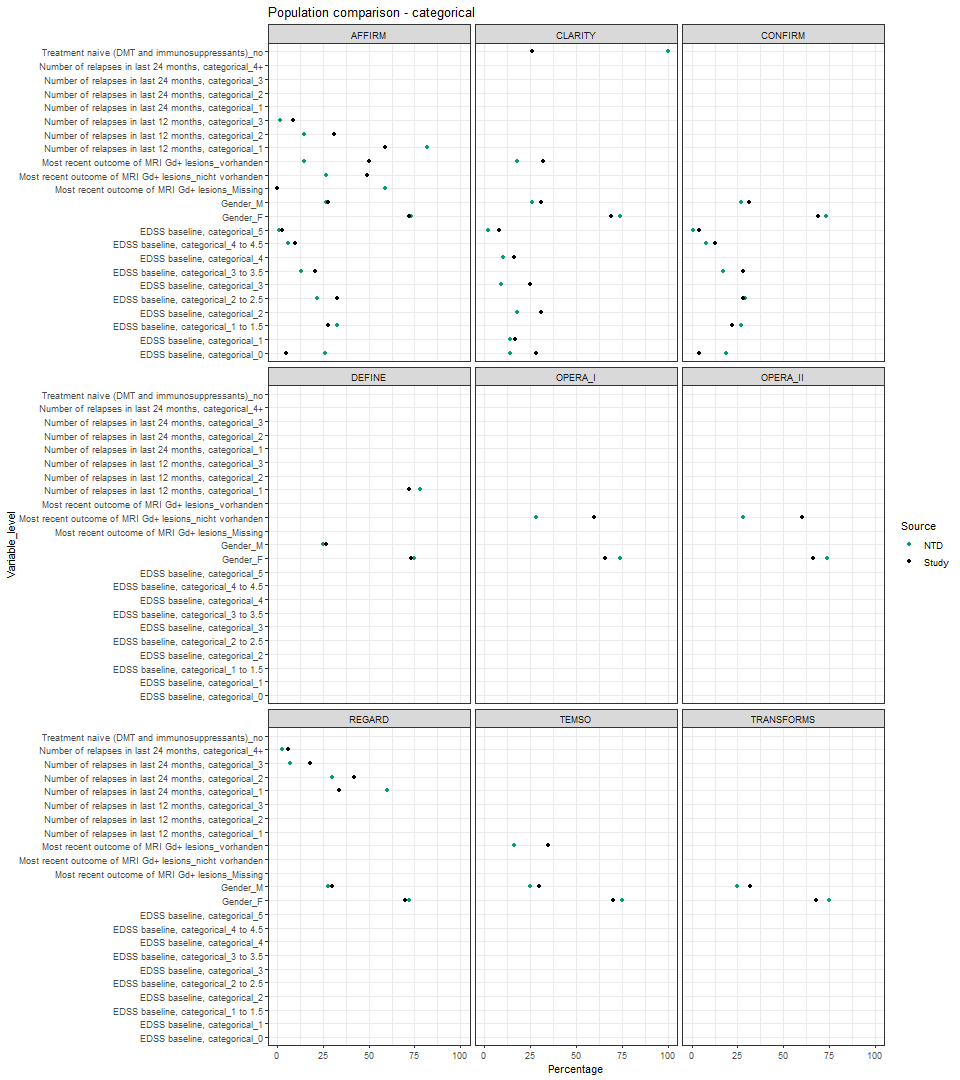


**Supplementary Figure 4:** Comparison of external study cohort and matched NTD MS registry cohort: categorical patient characteristics.

1. **Clinical meaningfulness**

| Case | Taken |  | Model | Dimethylfumarat | Fingolimod | Glatirameracetat | IF-beta1 | Natalizumab | Teriflunomide |
| --- | --- | --- | --- | --- | --- | --- | --- | --- | --- |
| Highest ranked therapy | FALSE |  | Relapse | 542 | 640 | 391 | 596 | 39 | 416 |
| Highest ranked therapy | TRUE |  | Relapse | 134 | 130 | 2 | 2 | 227 | 0 |
| First or second highest ranked therapy | FALSE |  | Relapse | 314 | 270 | 384 | 565 | 14 | 407 |
| First or second highest ranked therapy | TRUE |  | Relapse | 362 | 500 | 9 | 33 | 252 | 9 |
| One of the two least ranked therapies | FALSE |  | Relapse | 613 | 757 | 70 | 270 | 258 | 75 |
| One of the two least ranked therapies | TRUE |  | Relapse | 63 | 13 | 323 | 328 | 8 | 341 |
| Highest ranked therapy | FALSE |  | CDP | 379 | 682 | 351 | 588 | 148 | 401 |
| Highest ranked therapy | TRUE |  | CDP | 297 | 88 | 42 | 10 | 118 | 15 |
| First or second highest ranked therapy | FALSE |  | CDP | 217 | 529 | 233 | 491 | 89 | 369 |
| First or second highest ranked therapy | TRUE |  | CDP | 459 | 241 | 160 | 107 | 177 | 47 |
| One of the two least ranked therapies | FALSE |  | CDP | 564 | 397 | 360 | 477 | 217 | 152 |
| One of the two least ranked therapies | TRUE |  | CDP | 112 | 373 | 33 | 121 | 49 | 264 |

**Supplementary Figure 5:** number of patients in the DMT groups for the analyzes of clinical benefit of PHREND in treatment decisions in the NTD MS registry
